# Foods, Drugs, Instruments, &c.

**Published:** 1890-09-06

**Authors:** 


### A MEDICAL MUSEUM.

### FOODS, DRUGS, INSTRUMENTS, Etc.

Last week we gave a brief sketch of some of the principal exhibits which were shown at the "Museum" of the British Medical Association, whose annual meeting was held at Birmingham this year. This week we propose to make the description a little more complete by a brief notice of several other articles of great value, both as Foods and as Remedial Agents.

Apollinaris Water is a very old friend of the table, and claims as its constant patrons three of the most eminent and popular persons of the day—Her Majesty the Queen, Prince Bismarck, and Mr. H. M. Stanley. On the sober judgment of those three persons a great part of the world has long been accustomed to rely, in their own particular spheres. We need not for a moment doubt that they are well able to advise us about the taste and value of Apollinaris water. For our part, we are well content with the pure water of the spring or brook—when we can get it. But when there is any doubt upon the subject, as there generally is in London and on the Continent, we very much prefer the sparkling freshness and undoubted purity of Apollinaris.

Friedrichshall Water is a horse of another colour, or rather a fluid of another character. It belongs to the large and increasing class of aperient waters, and is one of the best known and most popular of its kind. Like many other things it has been improved as the result of competition. This might suggest the idea that it is an artificial water, and not an unsophisticated product of nature's laboratory. But that would be a mistake. The improvement does not consist in the method of manufacture, but of the taking of the water from the spring, and the bottling of it. There is high medical authority, of quite recent date, for the value of this water; but those who use it constantly are quite satisfied with the still higher authority of their own long experience. Of Friedrichshall it may emphatically be said that it is an excellent water.

Hunyadi Janos, though of more recent fame than Friedrichshall is a widely-known water of the mild aperient class, and well deserves its reputation.

Æsculap Water is of still more recent introduction, but it has probably outstripped all the other natural mineral waters in the race for popularity. On more than one occasion we have noticed it in these pages, and an extended experience of its palatability, and of its mild, painless, and certain action, enables us to confirm and emphasise all our most favourable previous opinions of its value. Of aperient mineral waters generally, it may be said that they constitute an absolutely indispensable part of the medical armamentarium.

From the pharmacy of nature to that of the chemist is a very short step in these scientific days. Perhaps before beginning with the inner man, we may say a word of the outer. Soap, which occupies a very inferior station in some countries, is quite among the royalties in our own. Messrs. Blondeau et Cie., of London, among other articles, showed their Vinolia Soap, of which we have before spoken in terms of high commendation in these pages. Of this soap, it may be said, that though it is costly, it is well worth the money. The same firm exhibited their Vinolia ointments and creams, which have won for themselves a wide-spread reputation. Their Vinolia powder was also shown. For the nursery, for rough skins, for sweating hands and feet, for mild eczemas, and for "weeping" surfaces generally, this powder is one of the best and safest known.

Messrs. Burroughs, Wellcome, and Company, the American chemists, who carry on their business at Snow Hill, were well to the fore with an extensive and beautiful selection of their unique preparations. Their "tabloids" and "tablets" are the despair of the pill makers. Many a poor dyspeptic has had reason to exclaim, "Now, blessings on the man who invented tabloids!" Cascara Sagrada Extract, that drug of vile taste, becomes almost a delicious confection when coated with sugar and reduced to a "tabloid." The same may be said of fifty other substances which have long enjoyed the unenviable reputation of "setting the teeth on edge." Messrs. Burroughs and Wellcome are very enterprising persons, and it cannot be denied that until within the last few years there was much room for originality and enterprise in the drug and physic world.

The Jeyes' Sanitary Compounds Company (Limited), of 43, Cannon Street, London, made an effective display with their various disinfectants and antiseptics. The well-known Jeyes' Fluid is as energetic as carbolic acid in its disinfecting action, and has the very marked advantage of being nonpoisonous and free from unpleasant smell. Messrs. Jeyes' also make disinfectant soaps, both for the toilet and for household and hospital use. They lay themselves out, in fact, for the manufacture of every kind of purifier—from refined powders and fluids for medical use to the coarser, stronger, and much cheaper substances which are employed for drains and other offensive accumulations. This company has established a high reputation, which it well deserves.

The Sanitas Company showed a great variety of their Sanitas Preparations, and notably their Kingzetts's Bactericides. Mr. Kingzett has given prolonged and intelligent study to the whole question of the destruction of bacteria; and his preparations, which are supplied by the Sanitas Company, are among the least offensive and most efficient of their kind.

The Sanitary Wood Wool Company, of Hatton Garden, London, are also manufacturers of purifying agents, though on somewhat different lines. They prepare all kinds of tissues, sheets, and cloths of an antiseptic character for surgical dressings and for use about the person. Their wood wool diapers are now made of the softness of cotton wool, and are much appreciated by those who use them. The sheets, which are specially designed for use in cases where antiseptic bedding is required, ensure the perfection of cleanliness and comfort, and are highly valued both by obstetric physicians and operating surgeons. The various articles prepared by this company are distinct acquisitions to the hospital and private nurses' repertory.

The Liverpool Lint Company, of the Mark Street Mills, Liverpool, showed a great variety of their excellent surgical dressings. The "wound pads," "splint pads," and "lints" of this company were much admired by practical surgeons, and quite as much by family practitioners. The pads of different kinds were particularly appreciated by those who attach importance to neatness and cleanliness in wound dressing and in the putting up of fractured limbs. The huge sheets of cotton wool of former days are rude and coarse compared with the thick, soft, and even pads of this company. They are made both antiseptic and plain, according as they are needed for simple fractures, or for open wounds. The firm also showed a great variety of bandages, styptic and surgeons' towels, and accouchement sheets. The excellence, neatness, and finish of all their articles were evidence of skilful manufacture and of conscientious determination to reach the highest level of good work.

The Foods of various kinds were, as we informed our readers last week, of a very high order of merit; and their manufacture in such variety marks an important advance in the character of the common necessaries of the sick room. Messrs. George Mason and Company exhibited a large number of their farinaceous and meat foods, put up in very appetising forms. They begin with a malted food for infants, and continue in an ascending scale until they reach an O. K. Bouillon for sportsmen and tourists. Included among their specialties are Peptonised Beef Tea, Nutrient Suppositories, Mason's Essence of Beef, and other well-known foods. The name of Mason is a sufficient guarantee of the excellence of the goods manufactured by the firm.

Cadbury's Cocoa was well represented, and its reputation is such that any words in its praise are altogether superfluous. Messrs. Cadbury may be congratulated on having a name and a fame which are known throughout the English-speaking world. The cocoa shown by this eminent firm to the members of the British Medical Association was undoubtedly an excellent article of food.

The Frame Food Company (Limited) showed specimens of their food, which is an extract of wheat bran. It is prepared in a variety of ways. The specialties of the company are Frame Food porridge, Frame Food infants' diet, Frame Food cocoa, &c. Chemical analysis shows that these foods contain a large percentage of wheat phosphates. The Frame Food may not only be used alone, but mixed with white flour and other meal, for making pastry, bread, and similar necessaries of the table. There is no doubt of the value of wheat bran, and the Frame Food Company have added one more to the nutritious and wholesome articles of diet by which the sick and the well of this generation are so greatly advantaged.

Nestle's Food for Infants was felt by every practitioner present to be an old familiar friend. There were very few medical men at Birmingham who could not have borne personal testimony to its worth. Many a thriving infant owes his chubby cheeks, his straight legs, and his happy smiles to Nestle's Food. There is no need either to describe or to praise it.

Dahl's Cakes are food of a different kind, and designed for a different purpose. They are called "dyspepsia cakes." It would be still more accurate to call them "constipation cakes." It would be difficult to find a better remedy for ordinary cases of constipation than Dahl's cakes. They contain no drug of any kind, either mineral or vegetable, but only coarsely ground meal. They are very wholesome, and not at all unpalatable. Of Dahl, as of many other little known persons, it may be said he is a benefactor of his kind.

Although we have selected certain names for special mention, we have by no means exhausted the long list of really excellent preparations of all kinds, which were shown by metropolitan and provincial manufacturers. Merely to give the names of such firms as Messrs. Richardson and Co., of Leicester; Loeflund and Co., Riddle, Alexander, and Co., J. F. and B. Macfarlane and Co., of Edinburgh; the Maltine Manufacturing Company; James Woolley, Sons, and Co., Manchester; Wyleys and Co., Coventry; Wilcox and Co., London; Warner and Co., Philadelphia and London; Seabury and Johnson; and other well-known firms, is to indicate very clearly to the profession that the annual meeting of the British Medical Association affords them an opportunity which is quite unique of becoming personally familiar with every new food and drug preparation which is put upon the market. It is much to be desired, in the interests both of doctors and their patients, that what we may call the "Peripatetic Museum" of the British Medical Association shall continue to attract in ever-increasing numbers the most eminent manufacturers and specialists of every civilised country.

